# Predicting podoplanin expression and prognostic significance in high-grade glioma based on TCGA TCIA radiomics

**DOI:** 10.1371/journal.pone.0325964

**Published:** 2025-06-24

**Authors:** Shengrong Long, Hongyu Xu, Mingdong Li, Lesheng Wang, Jiazhi Jiang, Wei Wei, Xiang Li

**Affiliations:** 1 Brain Research Center, Zhongnan Hospital of Wuhan University, Wuhan, China; 2 Department of Neurosurgery, Zhongnan Hospital of Wuhan University, Wuhan, China; 3 Department of Neurosurgery, First Affiliated Hospital of China Medical University, Shenyang, China; 4 Frontier Science Center for Immunology and Metabolism, Wuhan University, Wuhan, China; 5 Medical Research Institute, Wuhan University, Wuhan, China; 6 Sino-Italian Ascula Brain science Joint Laboratory, Wuhan University, Wuhan, China; First Affiliated Hospital of Anhui Medical University, CHINA

## Abstract

**Background:**

Podoplanin (PDPN) is a membrane glycoprotein implicated in tumor invasion and immune modulation in high-grade gliomas (HGGs). However, the non-invasive prediction of PDPN expression and its prognostic significance using radiomics remains unexplored.

**Materials and methods:**

This study used preoperative contrast-enhanced MRI T1WI data analyzed by gradient boosting machine to predict podoplanin (PDPN) expression and overall survival (OS) in HGG patients.

**Results:**

We retrospectively analyzed 89 HGG patients’ clinical data, MRI images, and RNA-seq profiles from TCIA. For each patient, 107 radiomics features were extracted from HGG subregions. The radiomics prognostic model was built using two selected features, glcm_Idmn and glcn_Idn. Through validation with external the REMBRANDT dataset (n=39), the model demonstrated great predictive performance for the PDPN expression levels and OS in HGG. The area under the curve of the ROC in the radiomics signature combined with clinical risk factors for the 1-year, 2-year, and 3-year OS rates in the TCIA-HGG were 0.799, 0.883, and 0.923, respectively. Gradient boosting machine using preoperative MRI T1WI and extracted radiomics features performed well in predicting the expression of PDPN and OS in HGG.

**Conclusions:**

Radiomics features extracted from MRI images can non-invasively predict PDPN expression and prognosis in HGG, offering a potential imaging biomarker for individualized clinical management.

## Background

Gliomas are one of the most common types of primary brain tumors [1,2]. The 2021 World Health Organization (WHO) classification of central nervous system tumors divides gliomas into grades 1–4, with grades 1–2 being low-grade gliomas (LGG) that typically have a relatively favorable prognosis [3]. Patients with LGG can achieve a median survival of up to 13 years following aggressive treatment [4,5]. Grade 3–4, high-grade gliomas account for approximately 80% of all primary intracranial malignancies, and it is estimated that 49.1% of these tumors are glioblastomas [6]. Several treatment options are available for high-grade gliomas, including surgery, radiotherapy, chemotherapy, and targeted therapies. However, patients with high-grade gliomas have a relatively low survival rate, with a reported five-year survival rate for glioblastoma of only 6.8% and a median overall survival of fewer than two years [7,8]. Current prognostic indicators for glioma, including clinical and pathological features, such as Ki67, isocitrate dehydrogenase (IDH), as well as imaging findings from computed tomography (CT) and magnetic resonance imaging (MRI) [9], insufficiently meet precision medicine needs. Rapid and non-invasive genotyping is urgently needed in clinical practice as targeted therapies are growing in cancer treatment [10].

Studies have shown that radiomics can assist in the early diagnose and stage of gliomas, and to assess tumor heterogeneity and microenvironments [11–14]. Radiomics is a non-invasive, dynamically measurable, and quantitative technique that provides a safer and more reliable way to follow up and predict patients’ health [15]. Furthermore, the technique can be beneficial when performing a brain tumor biopsy is impossible. The further advantage of radiomics is that it is fast and cost-effective, often using traditional clinical imaging methods [10,16]. Radiomics has been previously used to classify glioma subtypes [17]. The characteristics of glioma can also be classified using MRI-derived grey-scale invariant textures [18], five significant features [19], or wavelet-based features [17]. IDH mutation status, 1p/19q codeletion status, telomerase reverse transcriptase (TERT) promoter status, and O6-methylguanine-DNA methyltransferase (MGMT) methylation status have been used as key biomarkers to determine molecular subtypes [17]. As a diagnostic and prognostic biomarker in glioma, transcriptome subtyping may help improve treatment outcomes. These transcriptomic isoforms play crucial roles in glioma progression-free survival (PFS) and are implicated in resistance to temozolomide (TMZ) therapy [17]. Therefore, it is possible for non-invasive imaging biomarkers to identify transcriptomic subtypes of gliomas and to provide a more precise treatment.

In clinical practice, there is a lack of molecular markers that are sufficiently precise, even though current research has identified many prognostic genes. A transcriptomics-only approach is no longer sufficient to meet the development of clinical disciplines. Therefore, radiomics combined with transcriptomic analysis may provide novel insights into the diagnosis and treatment of gliomas, and a valid predictive marker for the personalized treatment of patients with gliomas.

Podoplanin (PDPN), a type I transmembrane mucin-like glycoprotein, is primarily expressed in lymphatic endothelial cells [20]. Recently, clinical evidence has suggested that PDPN plays an important role during epithelial-to-mesenchymal transition (EMT) in a wide range of cancers [21,22]. In various solid malignancies, including lung, head and neck squamous cell carcinomas and brain tumors [23–25], PDPN is highly expressed. PDPN may contribute to metastasis and infiltration of cancer cells and be a prognostic marker [23,26]. Mesenchymal glioblastoma has the worst prognosis of all glioblastoma subtypes and exhibits an upregulation in PDPN expression [27]. Therefore, PDPN may be a prognostic marker for high-grade gliomas, facilitating early diagnosis and prognosis prediction.

The gradient boosting machine algorithm is a member of the boosting algorithms family. It is an iterative, dependence-based algorithm that gradually reinforces the classifier as it iterates according to the user’s specified number of iterations. The algorithms reduce bias and variance in predictive models. A number of conditions can be predicted using gradient boosting machine, including cardiovascular events [28], sepsis [29], delirium [30], and readmission after lumbar laminectomy [31]. The clinical prognosis of patients with high-grade glioma was predicted non-invasively in this study by analyzing the expression level of PDPN using an enhanced MRI-based radiomics model, which was constructed using a gradient boosting machine in conjunction with transcriptomics. We propose a tool for accurately predicting prognosis in patients with high-grade glioma, which may be used as the basis for clinical diagnosis and treatment decisions.

## Materials and methods

### Ethics statement

This study publicly available, de-identified data from The Cancer Genome Atlas (TCGA), utilized only The Cancer Imaging Archive (TCIA), and REMBRANDT databases. Therefore, institutional ethical approval and informed consent were not required.

### The cancer genome atlas sample screening

**Fig 1:**
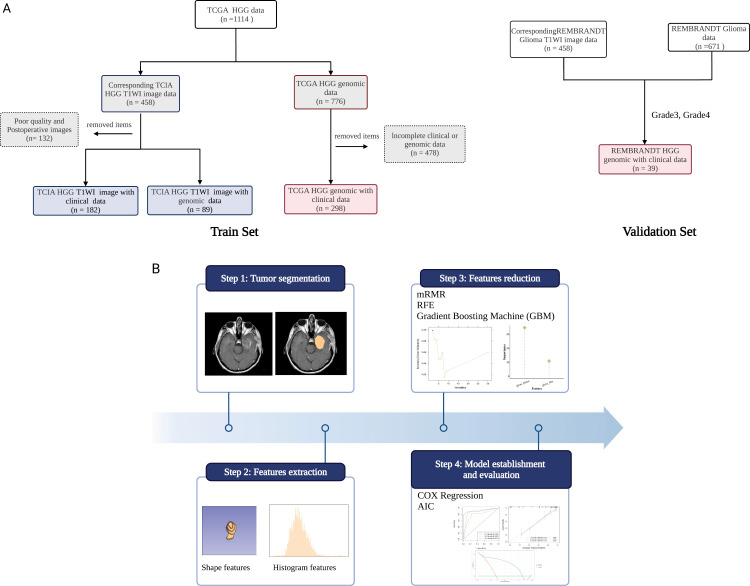
The radiomics workflow adopted in this study.

This study obtained all data and images for internal training and validation from the Cancer Genome Atlas (TCGA) and the Cancer Immunome Atlas (TCIA) databases. The transcriptome sequencing, and clinicopathological and follow-up data of patients with glioma were obtained from the TCGA database. The inclusion criteria were: 1. WHO grade 3–4 glioma samples; 2. primary glioma with sequencing data; and 3. available clinicopathological and follow-up data. Exclusion criteria were: 1. samples with missing pathological grade; 2. samples with missing survival data; 3. samples with survival time less than one month; and 4. samples with missing clinical data. In total, 298 cases of high-grade glioma from the TCGA database were included in this study. For external validation, data from the REMBRANDT dataset (n = 39) were used to assess the robustness and generalizability of the findings [32]. A detailed flowchart of the patient selection process is shown in Fig 1.

### Clinicopathological features and podoplanin (PDPN)

The cut-off values were determined using the survminer R package. Patients with high-grade gliomas were divided into high and low expression groups based on PDPN expression in RNA-sequencing to explore the correlation between the main variable PDPN and clinicopathological and other characteristics. Batch statistical analysis was performed and baseline information tables were drawn using the CBCgrps package.

### Intergroup variability analysis

For pre-processing, the PDPN expression data were converted to RNA-sequencing data in transcript per million (TPM) format and uniformly processed by the Toil process from UCSC XENA (https://xenabrowser.net/datapages/), and the glioblastoma multiforme and low-grade glioma of TCGA. The corresponding normal brain tissue data in the genotype-tissue expression (GTEx) project were extracted, and expression comparisons between the glioma group and normal brain tissue samples were performed using the linear models for microarray (limma) R package. The mapping of PDPN gene differential expression was completed by Grammar of Graphics plotting library (ggplot2) package.

### Analysis of subgroups and interaction tests

Cox proportional hazards model was used to investigate the relationship between one or more study factors and survival outcomes in high-grade glioma. Correlation analysis was performed using univariate and multifactorial Cox regression to compare the effect of the PDPN expression on patient prognosis in different subgroups of each covariate, including age, sex, glioma grade, IDH and mutation status, to determine whether PDPN was an independent prognostic factor for overall survival (OS). A corresponding HR > 1 indicated that the independent variable was a risk factor, while HR < 1 indicated that the independent variable was a protective factor. The cmprsk package and the forestplot package were used for the analysis of the results and forest plotting.

### Correlation analysis

Spearman’s rank correlation coefficient was used to perform the correlation analysis between the main variable PDPN and the clinical characteristics of high-grade glioma, including age, sex, glioma grade, IDH mutation status, and 1p19q co-deletion. The ggplot2 package was used to map correlations.

### Analysis of intergroup variability in PDPN and immune cell infiltration

The RNA-sequencing expression matrix of high-grade glioma samples was uploaded to the ImmuCellAI database (http://bioinfo.life.hust.edu.cn/ImmuCellAI#!/) and was used to assess the infiltration of various immune cells in glioma samples from the high and low PDPN expression groups. These immune cells included: NKT cells, CD4 + T cells, CD8 + T cells, and macrophages. The degree of immune cell infiltration between the PDPN high and low expression groups was also analyzed using the limma R package.

### GSVA enrichment analysis

Using the Gene Set Variation Analysis (GSVA) method [33], the expression matrix in TPM format of the expression levels between the high and low PDPN expression groups was transformed into a matrix of the expression levels of the gene sets between the two groups, and Hallmark (h.all.v7.5.1.symbols.gmt) and the Kyoto Encyclopedia of Genes and Genomes (KEGG) (c2. cp.kegg.v7.5.1.symbols.gmt) gene sets were downloaded from the MsigDB database [34]. Pathway enrichment scores were calculated for each sample to assess whether different pathways were enriched between samples. The limma R package was used to perform a differential analysis of the pathway enrichment scores of the high and low PDPN expression groups, to visualize the top 50 pathways with a threshold of t = |0|, and to perform an unsupervised classification to screen for specific biological significance in high-grade glioma samples with similar pathway activity.

### The cancer imaging archive (TCIA) sample screening

Medical imaging data, clinical and follow-up data of patients with high-grade glioma were obtained through the TCIA database (https://www.cancerimagingarchive.net/). We intersected the RNA-seq data from the TCGA database of patients with high-grade glioma with the data from the TCIA database of patients with high-grade glioma containing MRI images. TCIA allows for radiological feature extraction and model building for medical imaging, while TCGA sequencing data allows for further prognostic analysis of the PDPN gene and assessing the prognostic value of predictive models.

### Volume-of-interest (VOI) segmentation and evaluation of consistency

Image Pre-Processing was required prior to lesion delineation, with the N4ITK method used to correct bias field distortion, which can effectively reduce the potential effects of MR intensity heterogeneity in tissue areas [14]. Spatial resampling was then performed to resample the images to 3 × 3 × 3 mm^3^ isometric voxels. Finally, the Z-score transform was applied to normalize the image histology feature data. The above pre-processing reduces image noise and normalizes the intensity, reducing the variation in signal intensity of images acquired by different machines. Manual segmentation of the volume of interest (VOI) of each image was performed by an experienced radiologist and a neurosurgeon using 3D-Slicer software (version 4.10.2; https://www.slicer.org/). The intraclass correlation efficiency (ICC) was used to evaluate the consistency of the extracted imaging histological features based on the VOI outlined on each imaging slice by the two surgeons separately. Firstly, the radiologist outlined the VOI in all image samples. Using the random number table method, the neurosurgeon selected a random sample of images for VOI outline. Imaging histological features were extracted and the intraclass correlation coefficient (ICC) was calculated to assess intra-observer reliability. An ICC ≥ 0·80 was considered to be excellent agreement, 0.51–0.79 was moderate, and <0.50 was poor.

### Screening for imaging and histological features

Radiomics features extraction involves extracting data that are not observed by human vision and can represent the internal features of a tumor, including (i) morphological features; (ii) first-order features; (iii) second-order features; and (iv) higher-order features. The open source Pyradiomics package based on Python was used for feature extraction, and 107 features were extracted. The mRMR (minimum redundancy, maximum relevance) and RFE (recursive feature elimination) algorithms were used to filter the image features in the region of interest. First, feature screening (mRMR package) was performed by the mRMR algorithm to select the features with the most significant relevance to patient outcome (survival or death status). The minimum redundancy selection was applied to ensure minimal redundancy between features, and the top 30 features were selected for final modelling (S1 Data). These 30 features were then further filtered by the RFE algorithm, which ranked the features by importance (defined by how much each feature contributed to the model) and then recursively removed the features with the most negligible absolute weight from the feature set until the best features were obtained for the final modelling, based on the implementation of the R caret package [35].

### Construction of the prognosis model

In this study, the gradient boosting machine was used to train new weak classifiers based on the negative gradient information of the loss function of the current model and then combine the trained weak classifiers into the existing model in a cumulative form. By continuously improving the capability of the weak classifiers, a robust classifier with high performance to develop a prediction model for the filtered was developed. The modelling of the screened imaging features was used to predict the expression of the PDPN gene using the gradient boosting machine from the caret package [36,37].

### Feature selection and model construction

Gradient Boosting Machine algorithm is used to construct a prognosis prediction model based on radiology. Use the gbm method from caret package in R to perform model training. GBM iteratively trains weak classifiers based on the negative gradient of the loss function from the current model, and integrates them in an additive manner to form a strong ensemble classifier. This approach effectively reduces bias and variance, leading to a more robust and high-performing predictive model. Radiomics feature selection was performed in two sequential steps. First, the minimum redundancy maximum relevance (mRMR) algorithm was applied to identify the top 30 features most relevant to survival outcomes while minimizing redundancy among features. Second, recursive feature elimination (RFE) was employed to further refine the feature set by iteratively removing the least important features based on model performance.

The final GBM model was trained using the following key hyperparameters: number of trees = 100, interaction depth = 3, shrinkage = 0.1, and 10-fold cross-validation. These parameters were optimized through grid search to prevent overfitting and improve generalizability.

### Model evaluation and validation

The effectiveness of the model is evaluated in the training group and cross-validation (10-fold cross-validation). The evaluation metrics were accuracy (ACC), specificity (SPE), sensitivity (SEN), positive predictive value (PPV) and negative predictive value (NPV). The x-axis of the receiver operating characteristic (ROC) curve was the false positive rate (FPR) and the y-axis was the true positive rate (TPR). This study compared the discriminative accuracy of the model using the area under the curve (AUC) of the ROC. The recall curve (precision-recall, PR) and its AUC were analyzed using the modEvA package. The high sensitivity of the PR curve to positive samples makes it more suitable for assessing model performance by focusing on positive samples. The calibration of the imaging histology prediction model was evaluated by plotting calibration curves and performing Hosmer-Lemeshow goodness-of-fit tests (ResourceSelection package). The overall performance of the imaging histology prediction model was quantified by the Brier score (using the measures package), and the decision curve (DCA) was plotted to demonstrate the clinical benefit. The Delong test was used to compare the AUC values of the training and validation sets and test the model for over-fitting.

### Analysis of variability between model groups

The imaging histology model outputs a probabilistic radiomics score (RS) for predicting PDPN expression levels. The performance of the identification of imaging histology features was assessed by comparing whether there was a difference in RS between high and low PDPN subgroups using the Wilcoxon rank sum test. Exploring the potential association between imaging histology and PDPN expression levels in high-grade gliomas can be used to non-invasively predict the clinical prognosis of high-grade gliomas.

### The Cancer Imaging Archive-The Cancer Genome Atlas (TCIA-TCGA) consolidated data baseline table

The TCIA high-grade glioma image data intersected with the TCGA high-grade glioma clinical data to obtain the intersection sample. The RS of the intersecting sample was calculated by the established imaging model, and the RS was combined with the clinical data and classified into low/high dichotomous variables by calculating the RS cut-off value with the survminer package. The low/high RS grouping was used to create a clinical information table for the patient using the CBCgrps package.

### Survival analysis

KM curves were used to present the change in survival rates between the high and low RS groups. The horizontal axis on the KM curve represented time and the vertical axis represented the probability of survival. Log-rank tests were used to test the significance of survival between the two groups and the survminer package was used for statistical analysis.

### Construction and evaluation of the nomogram

In this study, the clinical variables were obtained by selecting the smallest AIC information statistic by constructing a multifactorial regression COX analysis with the Akaike information criterion (AIC), combined with RS. A column line graph nomogram including the following variables: RS, grade, chemotherapy, and radiotherapy was involved in the construction of the final model. The time dependent ROC curves of the nomogram were then plotted to illustrate the predictive power of the factors at different time points; calibration plots were plotted with the horizontal coordinate representing the predicted survival rate and the vertical coordinate representing the actual survival rate. The diagonal line represented the predicted probability equal to the actual probability, and the further the deviation from the diagonal line the greater the prediction error. The clinical benefit of the imaging histology prediction model was demonstrated by plotting the decision curve. The above evaluation methods are implemented through the ‘timeROC’ package, the ‘rms’ package and the ‘dcurves’ package.

## Results

### TCGA baseline information sheet

A total of 298 high-grade glioma samples from the TCGA database meeting the study criteria were categorized into two groups based on PDPN expression levels: high and low. The baseline patient characteristics, summarized in Table 1, revealed no significant differences between the high and low PDPN expression groups in terms of sex, chemotherapy, and radiotherapy variables (P > 0.05). In contrast, significant differences were observed in the distribution of age, tumor grade, IDH-status, chr-1q-19-codeletion, and MGMT-promoter-status between the two groups (P < 0.001).

**Table 1:** Patient clinical and pathological data are summarized for categorical variables based on podoplanin (PDPN) expression level.

Variables	Total (n = 298)	Low (n = 133)	High (n = 165)	p
Age, n (%)				< 0.001
~59	197 (66)	118 (89)	79 (48)	
60~	101 (34)	15 (11)	86 (52)	
Sex n (%)				0.24
Female	120 (40)	59 (44)	61 (37)	
Male	178 (60)	74 (56)	104 (63)	
Grade, n (%)				< 0.001
III	170 (57)	122 (92)	48 (29)	
IV	128 (43)	11 (8)	117 (71)	
IDH status, n (%)				< 0.001
Wildtype	169 (57)	8 (6)	161 (98)	
Mutant	129 (43)	125 (94)	4 (2)	
Chr_1p_19q codeletion, n (%)				< 0.001
Non-codel	248 (83)	83 (62)	165 (100)	
Codel	50 (17)	50 (38)	0 (0)	
MGMT promoter status, n (%)				< 0.001
Unmethylated/Unknown	118 (40)	15 (11)	103 (62)	
Methylated	180 (60)	118 (89)	62 (38)	
Chemotherapy, n (%)				0.466
NO	79 (27)	32 (24)	47 (28)	
YES	219 (73)	101 (76)	118 (72)	
Radiotherapy, n (%)				0.085
No	70 (23)	38 (29)	32 (19)	
Yes	228 (77)	95 (71)	133 (81)	

### PDPN intergroup variability and survival analysis

**Fig 2:**
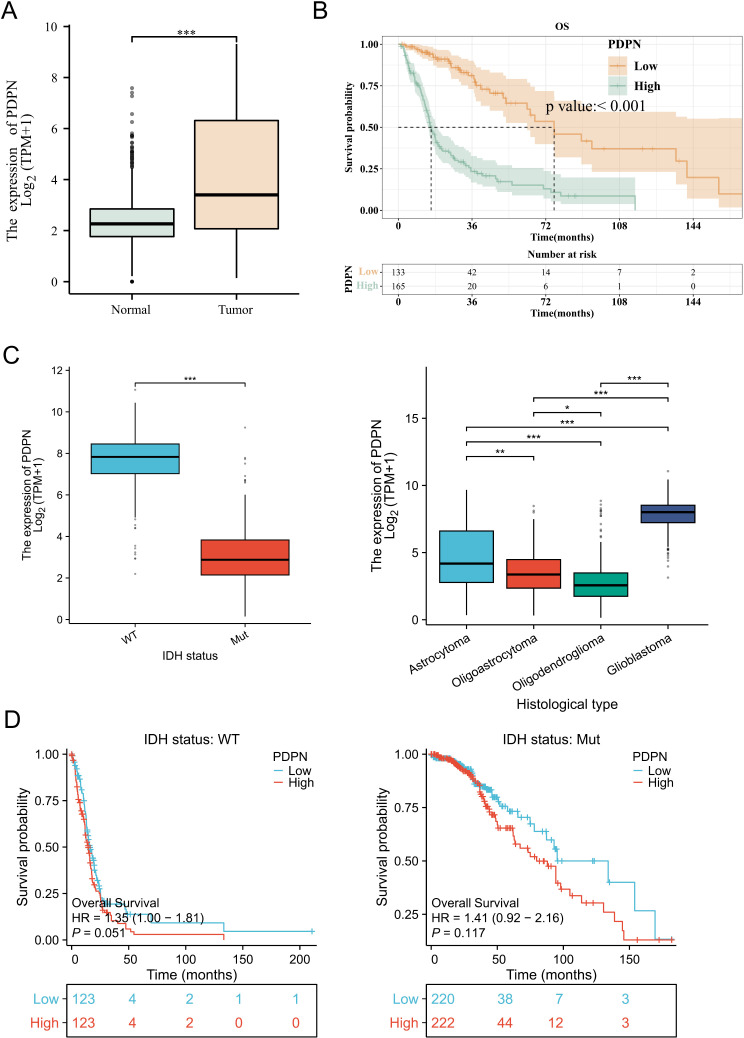
The comparison of clinical and survival data. (A) A comparison of the podoplanin (PDPN) expression level between the high-grade glioma and normal brain tissues. (B) The PDPN expression level Kaplan–Meier curve shows that patients with low expression levels have longer overall survival than patients with high expression; the log-rank test showed a significant difference (*p* < 0.001). (C) A comparison of the podoplanin (PDPN) expression level in subgroup. (D) Survival analyses according to PDPN expression in the IDH-WT and IDH-MUT subgroups.

Analysis of high-grade glioma samples from TCGA and normal tissue samples from GTEx indicated that PDPN was significantly higher in high-grade glioma tissue compared to normal tissues (p < 0.001) (Fig 2A). Survival analysis based on PDPN expression groups revealed that the median survival time for the PDPN low expression group was 76.1 months, while the median survival time for the PDPN high expression group was 16 months (S2 Data), with high PDPN expression being significantly associated with poor overall survival (OS) (P < 0.001) (Fig 2B). In addition, subgroup analyses demonstrated that PDPN expression was significantly higher in glioblastomas and IDH-wildtype gliomas compared to other subtypes (Fig 2C). Survival analyses further revealed that elevated PDPN expression was associated with worse overall survival specifically in the IDH-wildtype subgroup, whereas no significant prognostic effect of PDPN expression was observed in IDH-mutant gliomas (Fig 2D). These findings suggest that the prognostic relevance of PDPN may be influenced by IDH mutation status in glioblastoma.

### Univariate and multifactorial Cox regression with clinical correlation analysis

**Fig 3:**
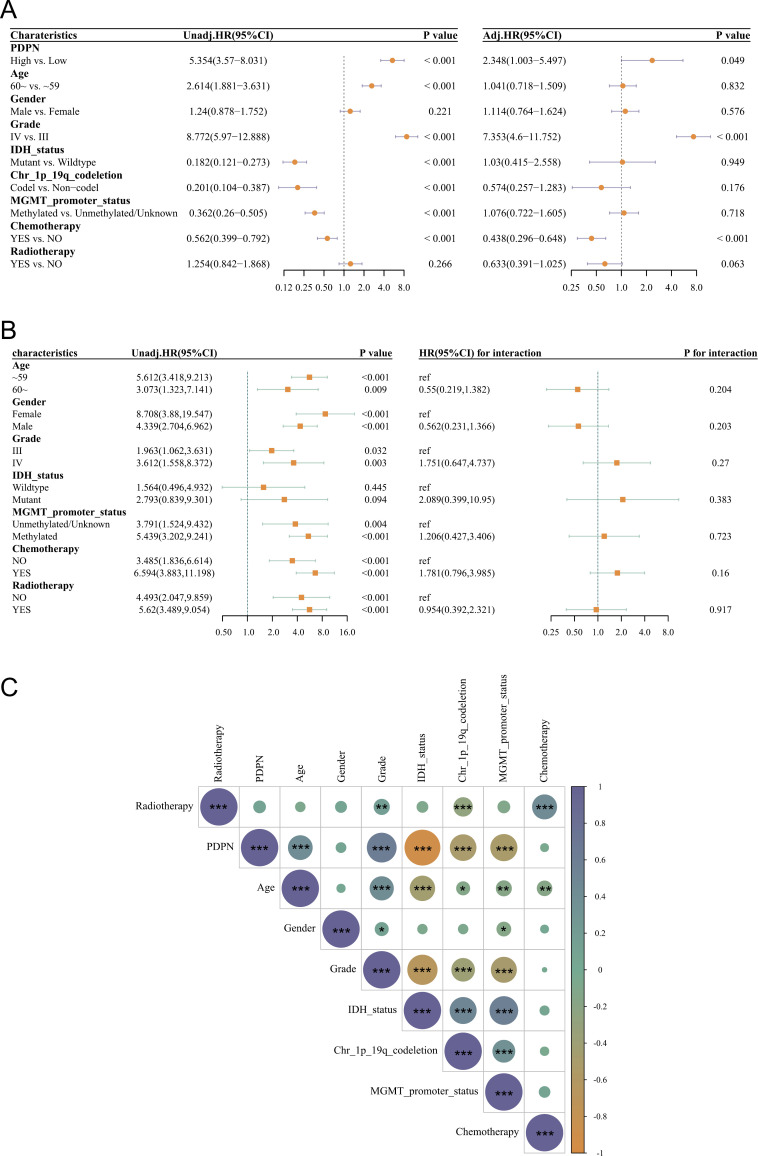
Association between podoplanin (PDPN) expression level and clinicopathological characteristics. (A) Associations between overall survival and clinicopathological characteristics using univariate and multivariable regression: PDPN. (B) Interaction test between PDPN expression level and clinicopathological characteristics. (C) The correlation between PDPN expression level and clinicopathological characteristics (*p < 0.05, **p < 0.01, ***p < 0.001).

Univariate Cox regression analyses performed on samples from both groups showed that the effect of PDPN on patient survival was statistically significant. High levels of PDPN expression increased the risk of high-grade gliomas and reduced patient survival time, predicting a worse prognosis (HR = 5.354, 95% CI 3.57–8.031). Other variables, age (HR = 2.614, 95% CI 1.881–3.631) and tumor grade (HR = 8.772, 95% CI 5.97–12.888) were also risk factors for poor prognosis in high-grade gliomas; conversely, some variables such as IDH-status, chr-1p-19q-codeletion, MGMT-promoter-status and chemotherapy delayed the development of high-grade gliomas and had a better prognosis and increased survival time. Adjusted for multifactorial cox regression, PDPN (HR = 2.348, 95% CI 1.003–5.497) remained a risk factor for poor prognosis in high-grade gliomas; while Grade (HR = 7.353, 95% CI 4.6–11.752) also acted as a risk factor. It also suggests a poor prognosis for high-grade gliomas (Fig 3A). In the subgroup analysis, elevated PDPN was a significant risk factor for OS in the female subgroup, HR (95% CI)=8.708(3.88,19.547), and in the male subgroup, elevated PDPN was a significant risk factor for OS, HR (95% CI)=4.339(2.704, 6.962); The P value for the interaction test was 0.203, indicating that there was no significant interaction of gender on the “association between PDPN and patient OS”, and the P value for the interaction test was 0.203, indicating that there was no significant interaction of gender on the poor prognosis of patients with high-grade gliomas. In the group of patients not undergoing chemotherapy, elevated PDPN was a significant risk factor for poor prognosis in patients with high-grade glioma, HR (95% CI) = 3.485 (1.836–6.614). In the group of patients undergoing chemotherapy, elevated PDPN was a significant risk factor for OS, HR (95% CI) = 6.594 (3.883–11.198) (Fig 3B). Correlation analysis of PDPN with the clinical characteristics of glioma patients showed that the main variable PDPN was significantly negatively correlated with IDH mutation status, chr-1q-19-codeletion and MGMT-promoter-status (p < 0.001), while it was significantly positively correlated with tumor grade and age (p < 0.05) (Fig 3C). Prior studies have suggested that IDH status may regulate PDPN expression in gliomas, and that IDH mutation stratification is clinically significant [38]. We assessed potential collinearity between PDPN expression and IDH mutation status by calculating variance inflation factors (VIF) in our multivariable Cox model; all VIF values were below 2, indicating minimal collinearity.

### Intergroup variability analysis of PDPN and immune cell infiltration

**Fig 4:**
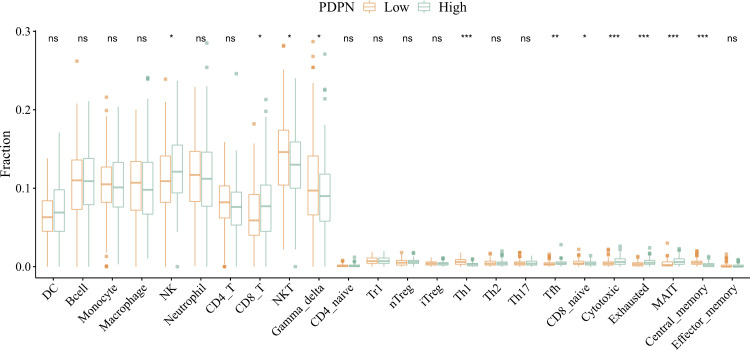
Estimation of tumour-infiltrating immune cells in high and low PDPN expression levels based on ImmuCellAI.

The immune cell infiltration in high-grade gliomas was analyzed, and inter-group variability analysis was performed. The results revealed that NKT cells and γδ T cells were significantly more infiltrated in the PDPN low expression group (P < 0.05), while infiltration of NK cells and CD8 + T cells was significantly greater in the PDPN high-expression group (P < 0.05) (Fig 4).

### GSVA between high and low groups for PDPN

**Fig 5:**
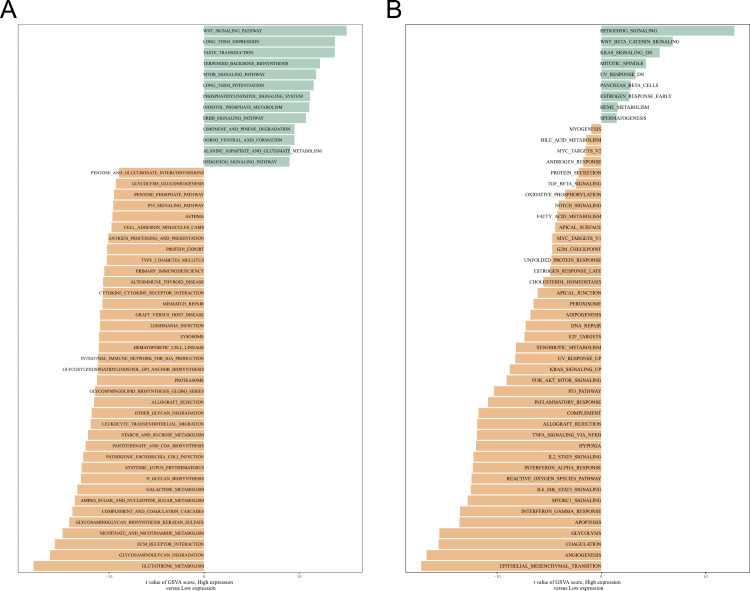
Gene Set Variation Analysis (GSVA) estimated differences in pathway podoplanin (PDPN) expression levels. (A) GSVA of the Hallmark Pathways further uncovered the differences between high and low PDPN expression levels. (B) Kyoto Encyclopedia of Genes and Genomes pathway analysis was performed to explore the potential mechanism between high and low PDPN expression levels using GSVA.

Differentially expressed gene sets between the high and low PDPN expression groups were analyzed for enrichment. In the KEGG gene set, the PDPN high-expression group was significantly enriched in signalling pathways such as WNT and MTOR, while the low-expression group was significantly enriched in signalling pathways such as glycolytic gluconeogenesis and P53 (Fig 5A). In the Hallmark gene set, the PDPN high-expression group was significantly enriched in the HEDGEHOG and WNT-Beta-linked protein pathways, whereas the low-expression group was significantly enriched in the epithelial-mesenchymal transition (EMT) and angiogenesis pathways (Fig 5B).

### TCIA image feature extraction and consistency evaluation

The TCGA raw signal data were intersected with the TCIA image data, yielding a sample size of n = 89. Radiologists outlined all 89 lesions, and surgeons randomly outlined 40 lesions. A total of 107 imaging features were extracted using the Pyradiomics package, and ICC was used to assess the consistency of the imaging features. The median ICC value was 0.955, with 96 features having an ICC value ≥0.80 (89.7% of all features), indicating high consistency.

### Screening and modelling of image features

**Fig 6:**
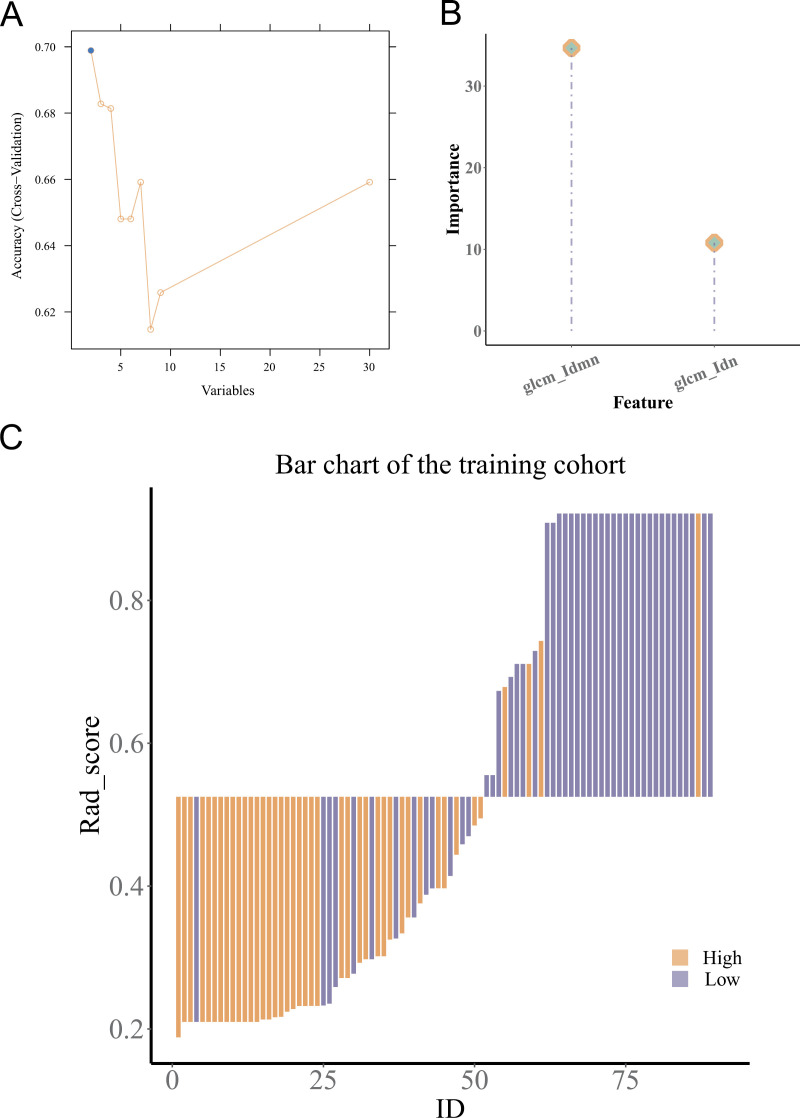
Construction of the radiomics model in high-grade glioma. (A) Recursive feature elimination analysis on feature radiomics reduction. (B) The model was constructed using the gradient boosting machine and the glcm_Idmn and glcm_Idn were selected. (C) The bar chart of the performance of the model prediction podoplanin expression level.

The extracted features were incorporated into subsequent feature screening. Two features were selected using the mRMR and RFE algorithm and modelled using the gradient boosting machine to predict the PDPN expression [39,40]. The diagram below illustrates the RFE screening process (Fig 6A). The figure indicates the importance of the features in the gradient boosting machine (Fig 6B). The visualization of the prediction results is shown in the figure (Fig 6C).

### TCIA-TCGA consolidated data

The 182 patients with HGG in the TCGA database included in the survival analysis were divided into a high RS expression group (n = 127) and a low RS expression group (n = 55) based on RS expression with a cut-off value of 0.2587. Clinical information for the patients is shown in Table 2. There were no significant differences in the distribution of sex, chemotherapy, or radiotherapy between the high and low RS expression groups (P > 0.05).

**Table 2:** Patient clinical and pathological data are summarized for categorical variables based on radiomics score (RS).

Variables	Total (n = 182)	Low (n = 55)	High (n = 127)	p
Age, n (%)				< 0.001
~59	112 (62)	47 (85)	65 (51)	
60~	70 (38)	8 (15)	62 (49)	
Sex, n (%)				0.16
Female	80 (44)	29 (53)	51 (40)	
Male	102 (56)	26 (47)	76 (60)	
Grade, n (%)				< 0.001
III	62 (34)	48 (87)	14 (11)	
IV	120 (66)	7 (13)	113 (89)	
IDH status, n (%)				< 0.001
Wildtype	134 (74)	18 (33)	116 (91)	
Mutant	48 (26)	37 (67)	11 (9)	
Chr_1p_19q_codeletion, n (%)				< 0.001
Non-codel	163 (90)	40 (73)	123 (97)	
Codel	19 (10)	15 (27)	4 (3)	
MGMT_promoter_status, n (%)				< 0.001
Unmethylated/Unknown	94 (52)	15 (27)	79 (62)	
Methylated	88 (48)	40 (73)	48 (38)	
Chemotherapy, n (%)				0.196
No	39 (21)	8 (15)	31 (24)	
Yes	143 (79)	47 (85)	96 (76)	
Radiotherapy, n (%)				0.182
No	34 (19)	14 (25)	20 (16)	
Yes	148 (81)	41 (75)	107 (84)	
OS, n (%)				< 0.001
0	57 (31)	37 (67)	20 (16)	
1	125 (69)	18 (33)	107 (84)	
OS time, median (Q1, Q3)	17.02 (9, 27.55)	22.57 (15.85, 39.68)	14.6 (7.38, 24.57)	< 0.001

### Model evaluation

**Fig 7:**
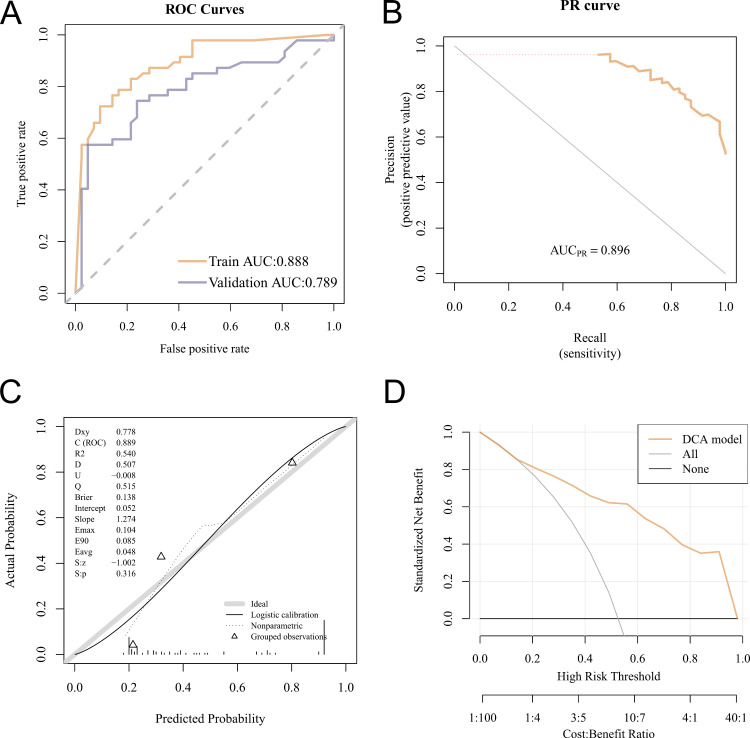
The evaluation the performance of radiomics model in different methods. (A) The receiver operating characteristic curve showing the performance in the training and validation cohort. (B) Precision-recall curve illustrating the classifier performance in the training cohort. (C) The Hosmer-Lemeshow test indicated that the calibration curves of the model were a good fit for the data. (D) Decision curve analysis for the model, established using the radiomics features from MRI T1WI sequences.

The constructed radiomics model demonstrated good prediction performance: the AUC value was 0.888 in the training set, and 0.789 in cross-validation (Fig 7A). The calibration curve and Hosmer-Lemeshow goodness-of-fit test showed good agreement between the predicted probability and the true value of the radiomics prediction model for whether a gene was highly expressed (p > 0.05) (Fig 7B, C). Decision curve analysis (DCA) showed the model has high clinical utility (Fig 7D). In addition, we used the REMBRANDT database to build an external validation cohort (S3 Data). The model showed similar strong predictive performance in the REMBRANDT cohort, with an AUC of 0.749 (S1A Fig). The calibration curve and the Hosmer-Lemeshow goodness-of-fit test again demonstrated good agreement between the predicted probabilities and true values for gene expression (P > 0.05). Additionally, DCA analysis confirmed that the model retains high clinical utility in the REMBRANDT cohort (S1B–D Fig).

### Inter-model group variability and survival analysis

**Fig 8:**
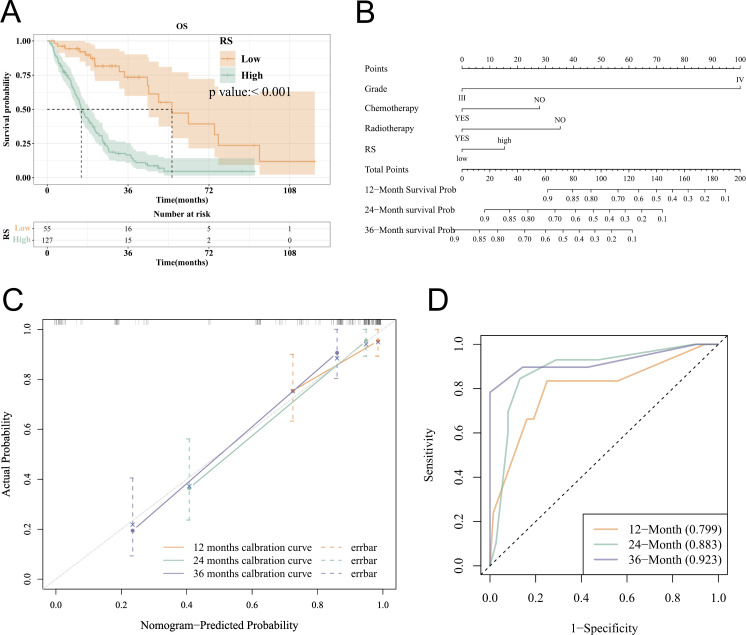
Evaluation of the performance of the model, combining radiomics score (RS) with clinicopathological characteristics. (A) The Kaplan–Meier curve based on RS shows that patients with low RS have longer overall survival than patients with high RS; the log-rank test showed a significant difference between the groups (*p* < 0.001). (B) The nomogram showed that the RS was combined with clinicopathological characteristics to determine the predictive performance of the model in HGG. (C) The calibration plot of this nomogram was developed, showing that the nomogram was well-calibrated for overall survival at 12, 24, and 36 months. (D) Receiver operating characteristic curve showing the performance of the model in the TCGA-HGG cohort.

The TCIA imaging data obtained were intersected with the TCGA clinical data (182 cases in total), and baseline information tables were drawn for each clinical variable using low/high RS as the grouping. This was followed by an analysis of variability between model groups. The results showed a significant difference in the distribution of Rad_score between the high and low gene groups, with high RS values in the PDPN high expression group, p < 0.001 (S1E Fig). The distribution of Rad_score in the REMBRANDT cohort was also consistent (S1F Fig). Survival analysis was performed after grouping and showed that the median survival time was 15.13 months in the high RS group and 55.53 months in the low RS group. The KM curve showed that high RS was significantly associated with the worse OS (p < 0.001) (Fig 8A).

### Nomogram plot and evaluation

Nomograms were used for plotting the probability of survival at 12 months, 24 months, and 36 months based on COX regression. As shown in the nomogram, RS, grade, chemotherapy, and radiotherapy were integrated into the final model (Fig 8B). As shown in the ROC curve, the AUC for the predictive power of the model for patient prognostic OS (36 months) has an AUC value of 0.923; the area under the curve for the predictive power of the model for patient prognostic OS (24 months) has an AUC value of 0.883; the calibration plot of the model shows that the curves at all times are around the diagonal, indicating a small error in prediction (Fig 8C); the DCA (24 months) shows that the model has high clinical utility within the threshold of 0.1 and 0.8 (Fig 8D).

## Discussion

Approximately 75% of gliomas in adults are estimated to be high-grade (WHO grades 3–4). Glioblastoma (WHO grade 4) accounts for over half (56.1%) of all gliomas and represents the most common and aggressive form [41]. Current research indicates that gliomas exhibit considerable genetic, epigenetic, and environmental heterogeneity [42–44]. The development of neuroimaging has provided new perspectives for the basic research, clinical diagnosis and treatment of gliomas. Several studies have demonstrated that MRI offers important clinical data for glioma patients, specifically, providing insights into tumor biology and genomic features, such as treatment response [45], tumor recurrence and p53 mutations [46]. Radiogenomics is a rapidly growing field that aims to identify imaging biomarkers for non-invasive genotyping by integrating imaging-derived parameters and genomic data. Radiogenomic genotyping presents considerable advantages: it captures tumor heterogeneity, can be performed repeatedly for treatment monitoring, and is applicable in cases where biopsy is not feasible [10].

Meanwhile, PDPN in neurons has been found to interact with CLCC-2 on platelets to promote platelet adhesion, aggregation, and secretion, thereby directing vascular maturation [47]. Interestingly, PDPN is associated with the progression and invasion of various cancers, including gliomas [48–51]. In glioma cells, the knockdown of PDPN leads to decreased proliferation, 2D migration, and invasion of collagen [49,52]. Studies have shown that the PI3K-AKT-AP-1 signalling pathway upregulates PDPN while EZH2 and oncogenic mutation of IDH1 downregulate PDPN, accompanied by changes in chromatin modification and DNA methylation. PDPN expression is strongly associated with a poor prognosis in gliomas [38]. Furthermore, PDPN has been proposed as a novel biomarker, chemotherapeutic target, and target for chimeric antigen receptor T-cell therapy and may be a potential form of immunotherapy for glioblastoma [27]. Therefore, combining PDPN expression levels in high-grade gliomas with MRI-based radiomics models allowed us to generate additional prognostic information to aid clinical decision-making.

This study presented a comprehensive machine learning methodology with feature selection to assess the prognosis of glioma patients [17]. This approach combines PDPN expression levels with radiomics data of high-grade glioma patients, incorporating the gradient boosting machine model into radiomic feature analysis and demonstrating significant correlations with both tumor heterogeneity and PDPN expression levels. By processing data from the TCGA and TCIA databases for statistical and imaging histological analysis, high PDPN expression was significantly associated with poor prognosis in patients with high-grade glioma through survival analysis as well as univariate and multivariate regression analyses. A non-invasive gradient boosting machine model was successfully developed by combining PDPN with contrast-enhanced MRI radiomic data to predict clinical prognosis. The prediction model was evaluated using AUC values, calibration curves and the Hosmer-Lemeshow goodness-of-fit test. Results achieved high and significant AUC values of 0.883 and 0.923 for predicting 24-month and 36-month prognosis, respectively (p < 0.05). DCA further demonstrated the high clinical utility.

Unlike other methods, the gradient boosting machine model can classify transcriptomic subtypes in glioma patients. Numerous studies have identified the gradient boosting machine model as one of the most effective models for glioma research [53,54]. Our study further demonstrates the importance of ensemble learning, particularly XGBoost, in imaging genomics. To provide a comparative view of the model effectiveness, we also compared it with previously reported models [55,56]. Our model achieved high accuracy and confidence, improving the average accuracy by approximately 10%.

Although the predictive model demonstrated robust performance in both the internal cohort (n = 89) and external validation cohort (REMBRANDT, n = 39), we acknowledge that the relatively limited sample size may affect the generalizability of our findings. To mitigate this, we employed rigorous 10-fold cross-validation and tested the model using an independent dataset to enhance its reliability. Nevertheless, future studies incorporating larger and more diverse patient populations from multiple centers are warranted to further validate the model’s applicability across different clinical settings and imaging protocols. Notably, interactions between key molecular markers (e.g., IDH mutation, MGMT promoter methylation) and imaging features were not fully explored due to current dataset constraints and limited subgroup sample sizes. Future studies incorporating larger, multi-center cohorts will enable more comprehensive stratified analyses and facilitate comparisons with standardized assessment systems such as RANO criteria, thereby improving clinical interpretability and utility.

In addition, to better understand the biological underpinnings of our radiomics signature, we examined the two selected grey‑level co‑occurrence matrix (GLCM) features, glcm_Idmn (inverse difference moment normalized) and glcm_Idn (inverse difference normalized). Both metrics quantify local textural homogeneity versus heterogeneity: higher values indicate more uniform image intensities, whereas lower values reflect greater heterogeneity and sharper grey‑level transitions. Tumor regions with high spatial heterogeneity often correspond to irregular vascularization, necrosis or infiltrative growth fronts, all of which can modulate the local immune microenvironment. Indeed, recent radiogenomic studies have shown that GLCM‑derived heterogeneity correlates with increased lymphocyte and macrophage infiltration in gliomas and with hypoxia‑induced immunosuppressive niches [57,58]. Therefore, in our model the discriminatory power of glcm_Idmn and glcm_Idn likely stems from their ability to capture subtle variations in tumor architecture that mirror underlying immune cell spatial patterns. Future work will aim to directly co‑register histopathology or immunohistochemistry maps with radiomic outputs to validate this radiographic–biological link. Additionally, we recognize the potential impact of manual VOI segmentation bias on radiomic feature stability. Although high inter-observer agreement was achieved (median ICC = 0.955), manual delineation can still introduce variability, and future implementation of semi-automatic or deep learning-based segmentation methods may further improve consistency. Furthermore, while our study focused on contrast-enhanced T1WI sequences, integrating multi-sequence imaging (e.g., T2, FLAIR, DWI) could provide a more comprehensive representation of tumor heterogeneity and is an important direction for future radiogenomic research. In this study, the radiomics model was developed using imaging data from a single center with standardized acquisition protocols and MRI platforms. However, radiomic features have been shown to be sensitive to variations in scanner type, acquisition parameters, and reconstruction settings, which may impact model robustness and reproducibility in external multi-center or multi-sequence settings [59]. In particular, differences in imaging contrast, spatial resolution, and noise across MRI sequences can introduce significant variability in extracted features, potentially affecting model generalizability. As such, further validation of the proposed model in independent, multi-center cohorts using heterogeneous imaging protocols is essential. Future work should also consider implementing harmonization techniques—such as intensity normalization or ComBat adjustment—to mitigate inter-site and inter-scanner variability and enhance model transferability across clinical settings.

With the increasing openness of public datasets, the predictive performance of radiomics-based machine learning models is expected to improve substantially, especially for prognostic analyses of gliomas. This gradient boosting machine model is anticipated to have considerable impact on prognostic assessment and treatment selection for patients with high-grade gliomas.

## Supporting information

S1 Fig Evaluation of the performance of the model. (A) The receiver operating characteristic curve showing the performance in the external validation cohort. (B) Precision-recall curve illustrating the classifier performance in the external validation cohort. (C) The Hosmer-Lemeshow test indicated that the calibration curves of the model were a good fit for the data. (D) Decision curve analysis for the model. (E) The distribution of Rad_score between the high and low expression groups.
(TIF)

S1 Data Selected radiomic features labels used in model training. (CSV)

S2 Data Survival data and IDH/MGMT mutation status for all included patients. (CSV)

S3 Data PDPN expression labels and tumor grade used in REMBRANDT cohorts. (CSV)
