# Supplementary figures and images for: Predicting podoplanin expression and prognostic significance in high-grade glioma based on TCGA TCIA radiomics
Source: PLoS One. 2025 Jun 24;20(6):e0325964. doi: 10.1371/journal.pone.0325964 (PMC12186906; doi:10.1371/journal.pone.0325964)

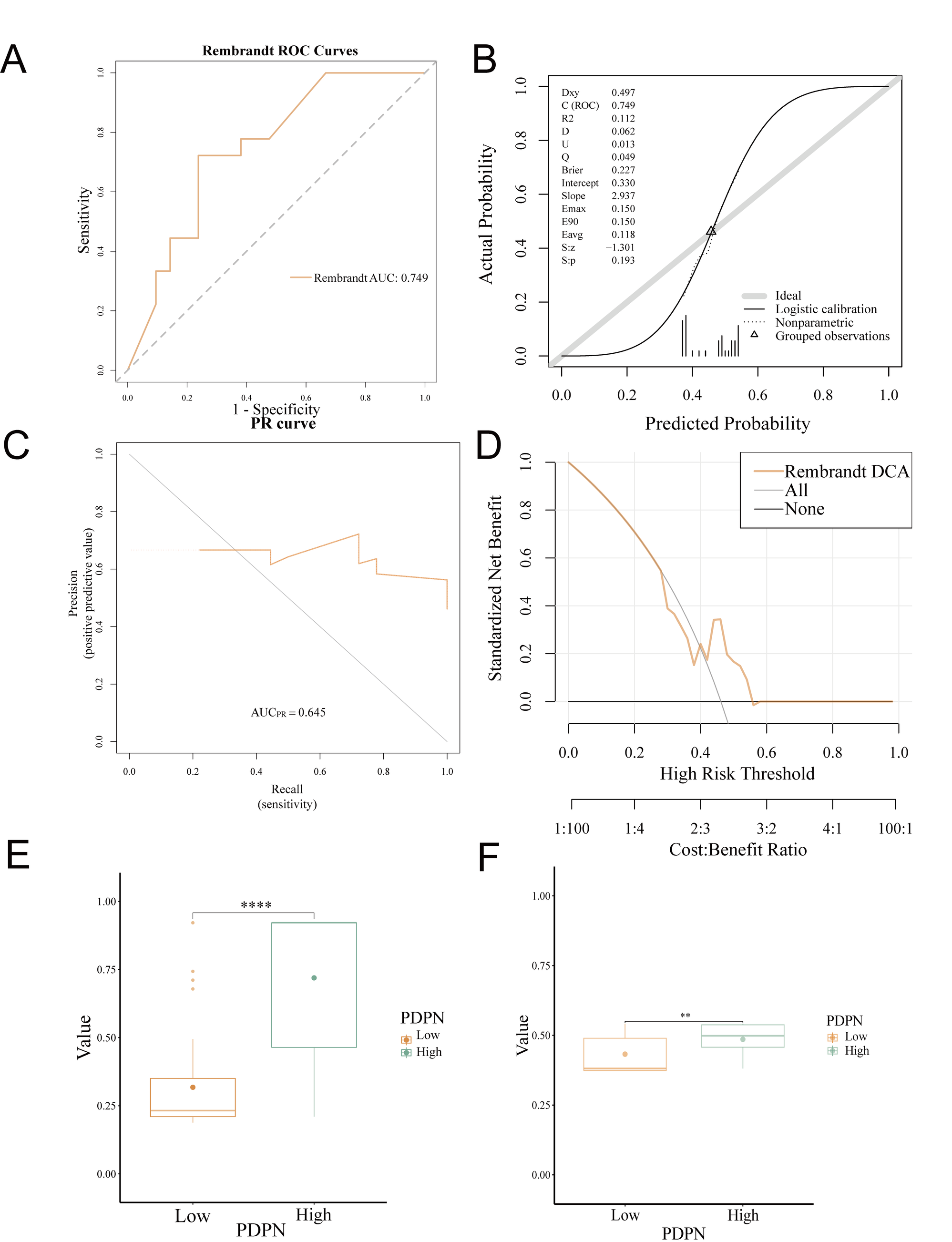

Supplement: S1 Fig — (A) The receiver operating characteristic curve showing the performance in the external validation cohort. (B) Precision-recall curve illustrating the classifier performance in the external validation cohort. (C) The Hosmer-Lemeshow test indicated that the calibration curves of the model were a good fit for the data. (D) Decision curve analysis for the model. (E) The distribution of Rad_score between the high and low expression groups. (TIF) [file pone.0325964.s001.tif]
